# Supplementary material for: Whole-Genome Bisulfite Sequencing Reveals a Role for DNA Methylation in Variants from Callus Culture of Pineapple (Ananas comosus L.)
Source: Genes (Basel). 2019 Nov 1;10(11):877. doi: 10.3390/genes10110877 (PMC6895883; doi:10.3390/genes10110877)
Supplement: Supplementary file 1 [file genes-10-00877-s001.zip › supplementary files/Table S1.docx]

TableS1. Summary of sequencing results and read alignment

|  | **Clean reads** | **Mapped reads** | **Mapped ratio (%)** | **sequencing depth (X)** |
| --- | --- | --- | --- | --- |
| CK | 137,934,206 | 118,658,828 | 86.03 | 46.61 |
| WS | 133,967,826 | 114,573,183 | 85.52 | 45.00 |
| YS | 220,440,122 | 185,127,874 | 83.98 | 72.71 |
| GS | 122,918,554 | 105,452,145 | 85.79 | 41.42 |
| LS | 124,401,174 | 103,889,938 | 83.51 | 40.80 |
| TP | 113,400,154 | 94,469,401 | 83.31 | 37.10 |
