# Supplementary material for: Whole-Genome Bisulfite Sequencing Reveals a Role for DNA Methylation in Variants from Callus Culture of Pineapple (Ananas comosus L.)
Source: Genes (Basel). 2019 Nov 1;10(11):877. doi: 10.3390/genes10110877 (PMC6895883; doi:10.3390/genes10110877)
Supplement: Supplementary file 1 [file genes-10-00877-s001.zip › supplementary files/Table S2.docx]

Table S2 The statistical results of C locus coverage in chromosomes of 6 samples

|  | C | | | CG | | | CHG | | | CHH | | |
| --- | --- | --- | --- | --- | --- | --- | --- | --- | --- | --- | --- | --- |
| Sample | Total sites | Coverage sites | Coverage rate(%) | Total sites | Coverage sites | Coverage rate(%) | Total sites | Coverage sites | Coverage rate(%) | Total sites | Coverage sites | Coverage rate(%) |
| CK | 117,404,024 | 96,408,751 | 82.12 | 19,229,458 | 15,610,321 | 81.18 | 16,596,772 | 13,833,590 | 83.35108779 | 81,577,766 | 66,964,840 | 82.09 |
| WS | 117,404,024 | 96,463,691 | 82.16 | 19,229,458 | 15,477,090 | 80.49 | 16,596,772 | 13,773,630 | 82.98981272 | 81,577,766 | 67,212,969 | 82.39 |
| YS | 117,404,024 | 98,681,231 | 84.05 | 19,229,458 | 15,904,064 | 82.71 | 16,596,772 | 14,075,643 | 84.809522 | 81,577,766 | 68,701,524 | 84.22 |
| GS | 117,404,024 | 95,418,029 | 81.27 | 19,229,458 | 15,227,427 | 79.19 | 16,596,772 | 13,600,803 | 81.94848372 | 81,577,766 | 66,589,799 | 81.63 |
| LS | 117,404,024 | 96,555,327 | 82.24 | 19,229,458 | 15,493,818 | 80.57 | 16,596,772 | 13,788,543 | 83.07966754 | 81,577,766 | 67,272,964 | 82.46 |
| TP | 117,404,024 | 95,467,218 | 81.31 | 19,229,458 | 15,264,939 | 79.38 | 16,596,772 | 13,630,998 | 82.13041669 | 81,577,766 | 66,571,279 | 81.60 |
