# Supplementary material for: Whole-Genome Bisulfite Sequencing Reveals a Role for DNA Methylation in Variants from Callus Culture of Pineapple (Ananas comosus L.)
Source: Genes (Basel). 2019 Nov 1;10(11):877. doi: 10.3390/genes10110877 (PMC6895883; doi:10.3390/genes10110877)
Supplement: Supplementary file 1 [file genes-10-00877-s001.zip › supplementary files/illustrated of figure S.pptx]

## Slide 1
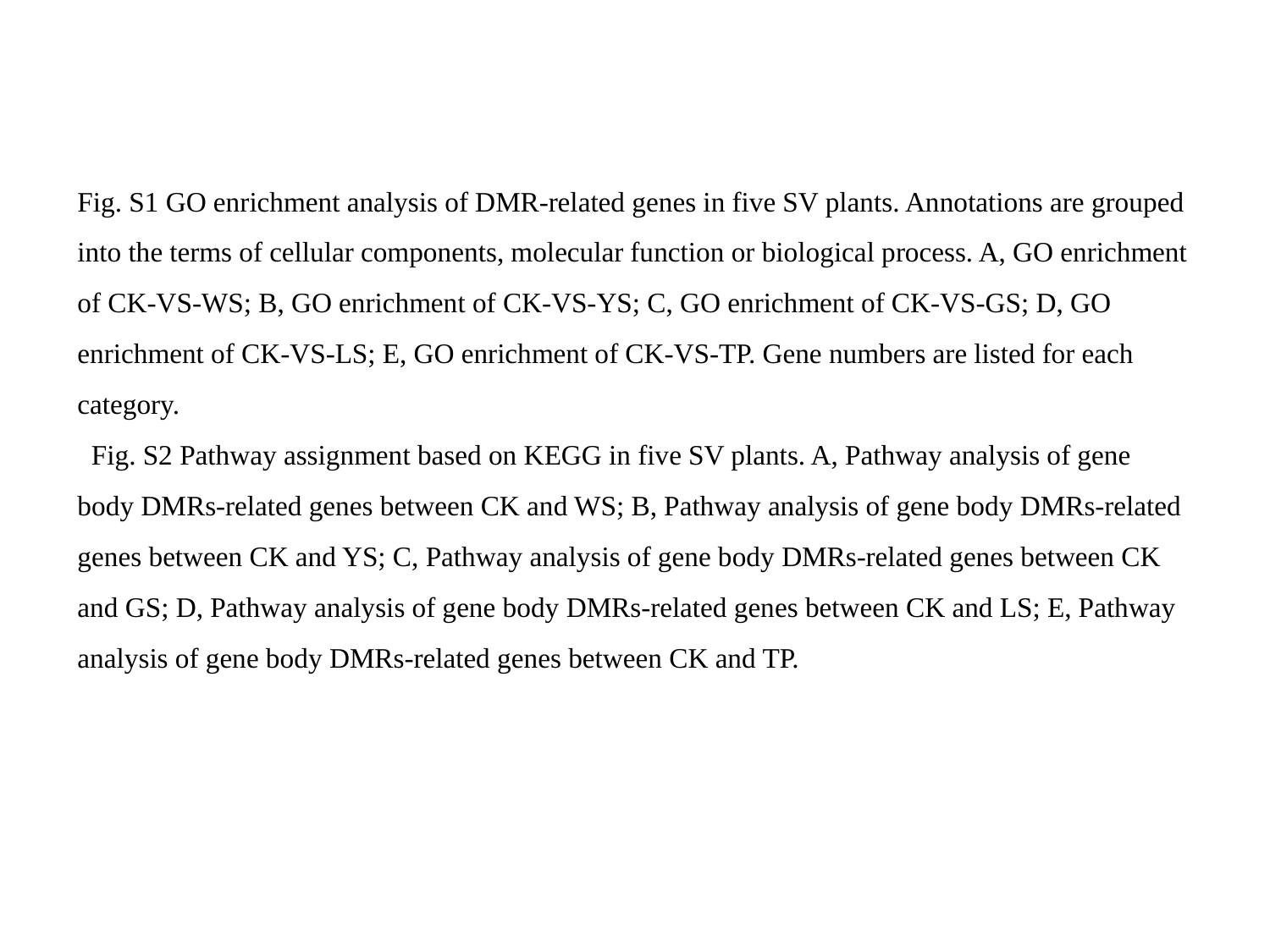

Fig. S1 GO enrichment analysis of DMR-related genes in five SV plants. Annotations are grouped into the terms of cellular components, molecular function or biological process. A, GO enrichment of CK-VS-WS; B, GO enrichment of CK-VS-YS; C, GO enrichment of CK-VS-GS; D, GO enrichment of CK-VS-LS; E, GO enrichment of CK-VS-TP. Gene numbers are listed for each category.
 Fig. S2 Pathway assignment based on KEGG in five SV plants. A, Pathway analysis of gene body DMRs-related genes between CK and WS; B, Pathway analysis of gene body DMRs-related genes between CK and YS; C, Pathway analysis of gene body DMRs-related genes between CK and GS; D, Pathway analysis of gene body DMRs-related genes between CK and LS; E, Pathway analysis of gene body DMRs-related genes between CK and TP.
