# Supplementary figures and images for: Whole-Genome Bisulfite Sequencing Reveals a Role for DNA Methylation in Variants from Callus Culture of Pineapple (Ananas comosus L.)
Source: Genes (Basel). 2019 Nov 1;10(11):877. doi: 10.3390/genes10110877 (PMC6895883; doi:10.3390/genes10110877)

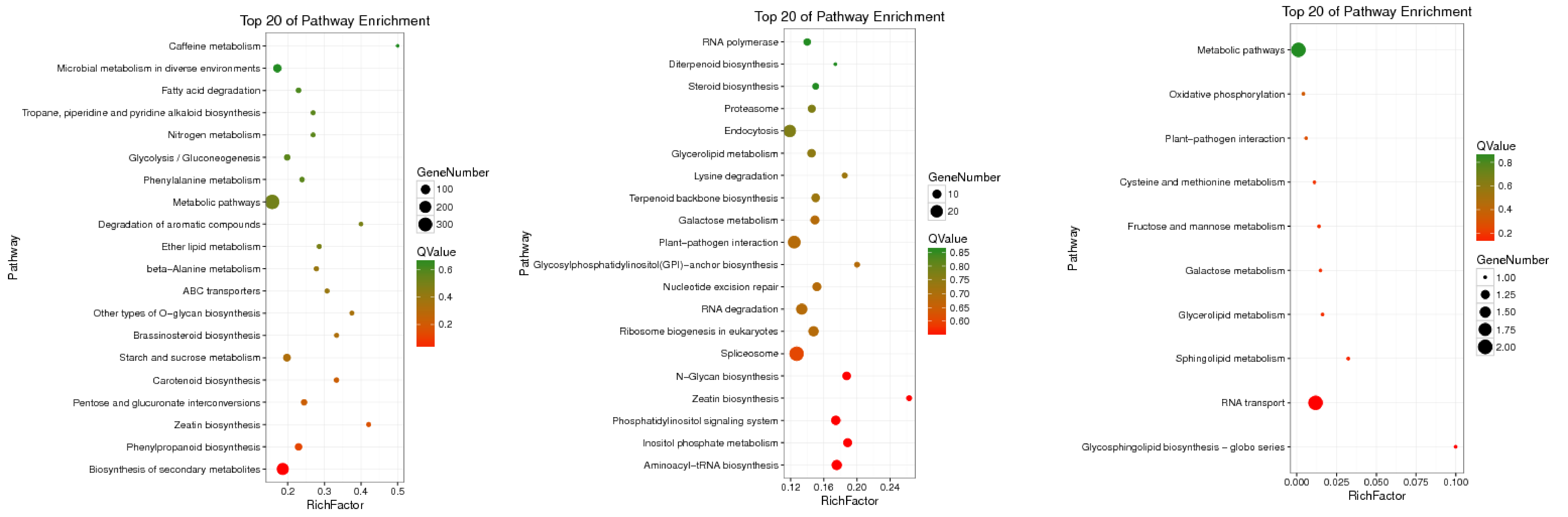

Supplement: Supplementary file 1 [file genes-10-00877-s001.zip › supplementary files/Fig S2A.jpg]

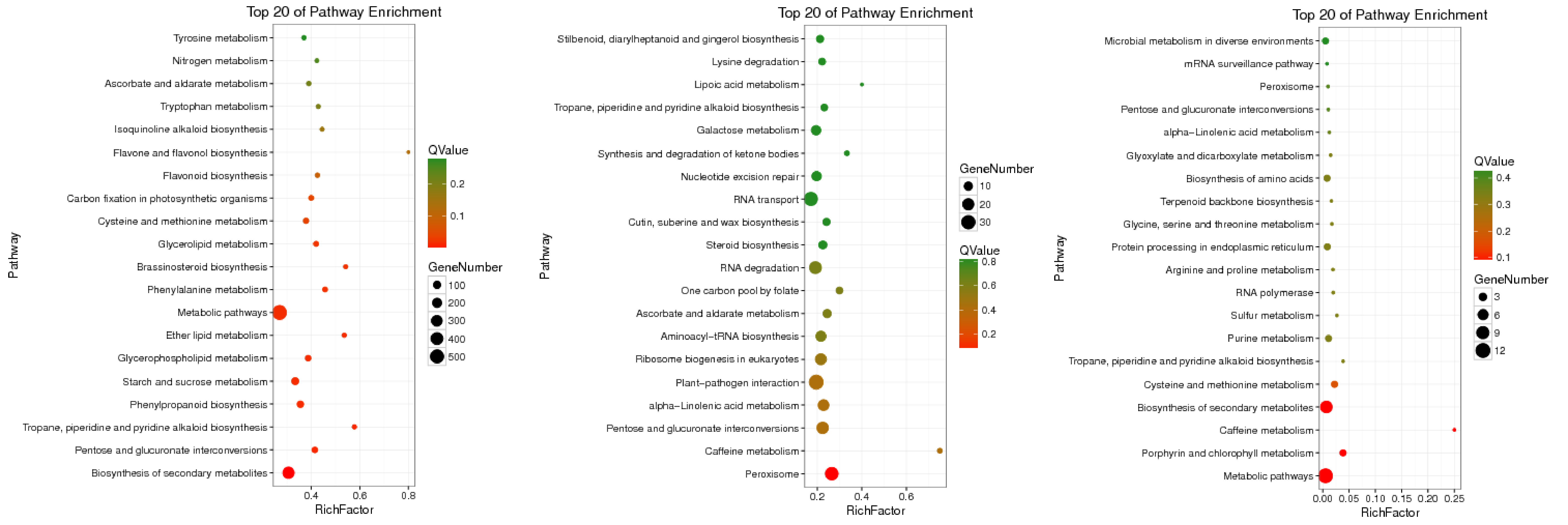

Supplement: Supplementary file 1 [file genes-10-00877-s001.zip › supplementary files/Fig S2B.jpg]

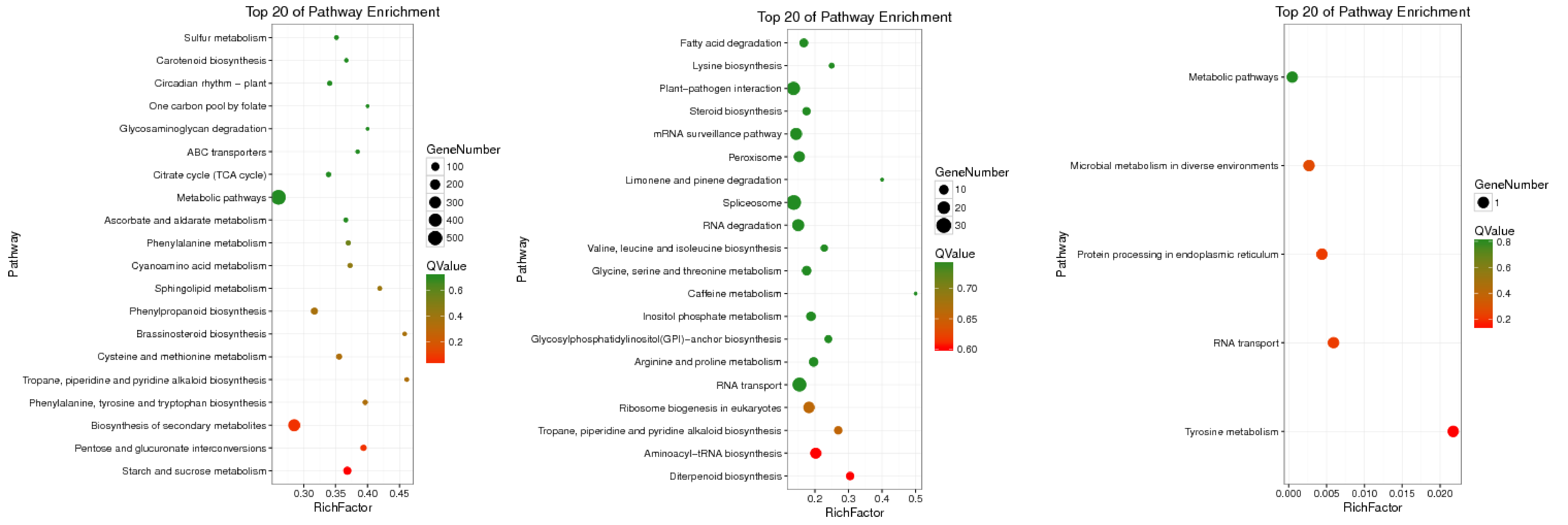

Supplement: Supplementary file 1 [file genes-10-00877-s001.zip › supplementary files/Fig S2C.jpg]

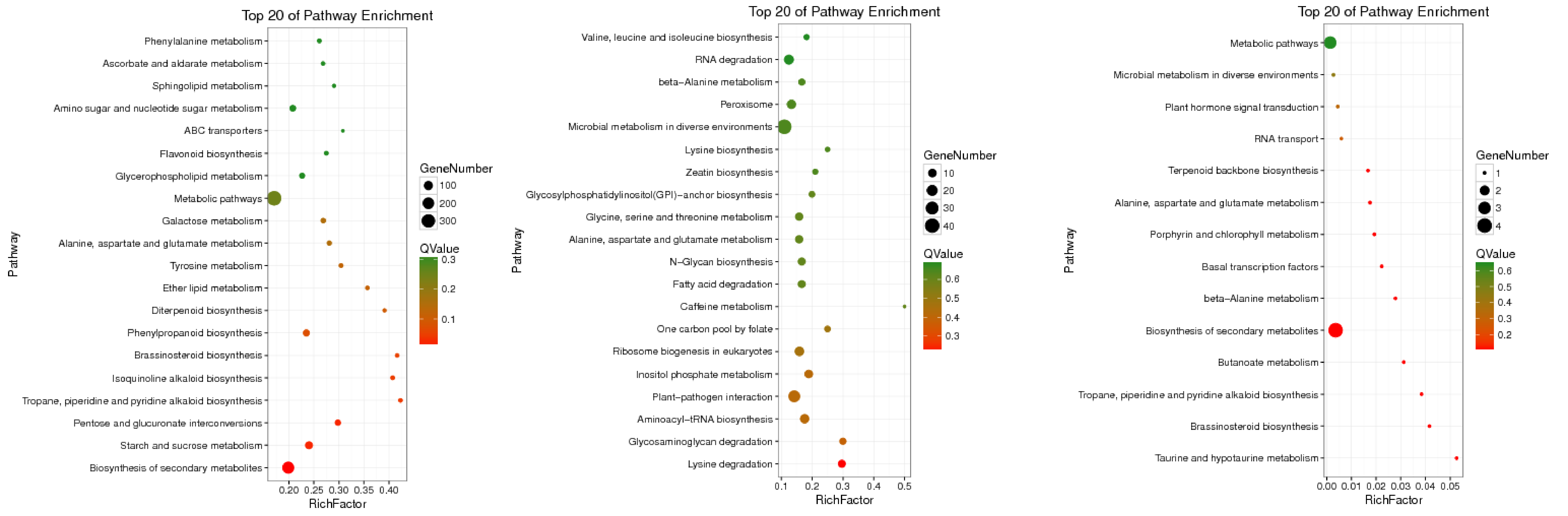

Supplement: Supplementary file 1 [file genes-10-00877-s001.zip › supplementary files/Fig S2D.jpg]

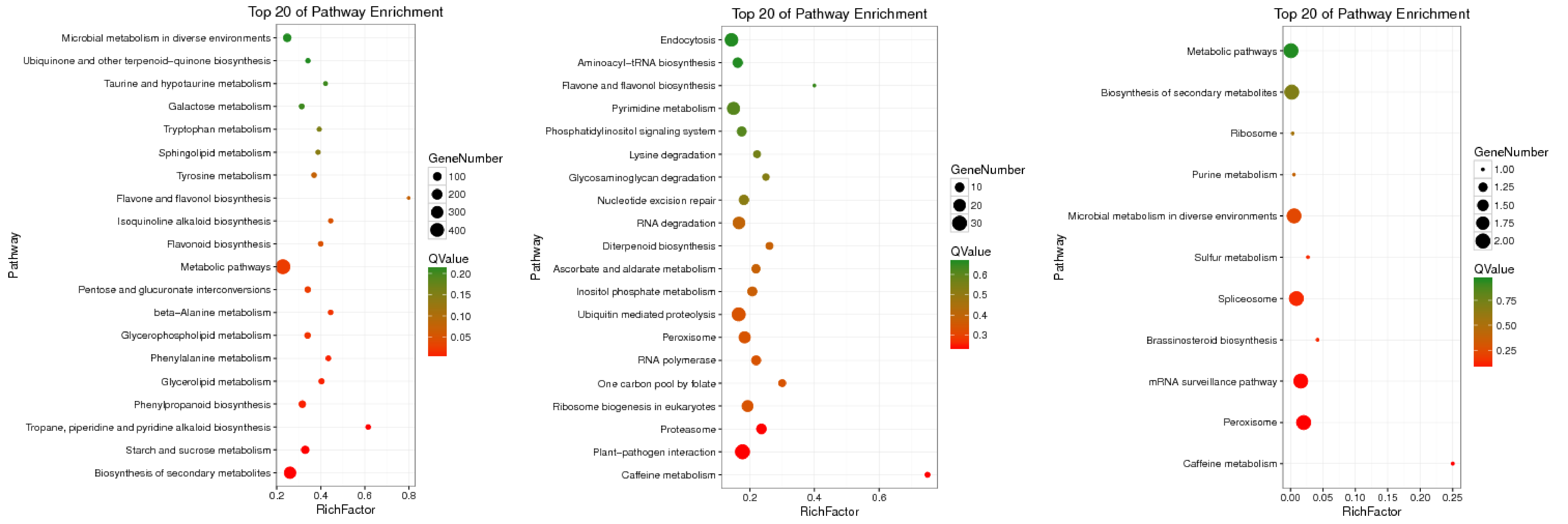

Supplement: Supplementary file 1 [file genes-10-00877-s001.zip › supplementary files/Fig S2E.jpg]
